# Toward the 2020 goal of soil-transmitted helminthiasis control and elimination

**DOI:** 10.1371/journal.pntd.0006606

**Published:** 2018-08-14

**Authors:** Sören L. Becker, Harvy Joy Liwanag, Jedidiah S. Snyder, Oladele Akogun, Vicente Belizario., Matthew C. Freeman, Theresa W. Gyorkos, Rubina Imtiaz, Jennifer Keiser, Alejandro Krolewiecki, Bruno Levecke, Charles Mwandawiro, Rachel L. Pullan, David G. Addiss, Jürg Utzinger

**Affiliations:** 1 Swiss Tropical and Public Health Institute, Basel, Switzerland; 2 University of Basel, Basel, Switzerland; 3 Institute of Medical Microbiology and Hygiene, Saarland University, Homburg/Saar, Germany; 4 Ateneo School of Medicine and Public Health, Ateneo de Manila University, Metro Manila, the Philippines; 5 Children Without Worms, The Task Force for Global Health, Decatur, Georgia, United States of America; 6 Rollins School of Public Health, Emory University, Atlanta, Georgia, United States of America; 7 Soil-Transmitted Helminthiasis Advisory Committee, Decatur, Georgia, United States of America; 8 Modibbo Adama University of Technology, Yola, Nigeria; 9 College of Public Health, University of the Philippines, Manila, the Philippines; 10 Department of Environmental Health, Emory University, Atlanta, Georgia, United States of America; 11 Department of Epidemiology, Biostatistics, and Occupational Health, McGill University, Montreal, Quebec, Canada; 12 Instituto de Investigaciones en Enfermedades Tropicales, Universidad Nacional de Salta, Oran, Argentina; 13 Department of Virology, Parasitology, and Immunology, Faculty of Veterinary Medicine, Ghent University, Ghent, Belgium; 14 Kenya Medical Research Institute, Nairobi, Kenya; 15 London School of Hygiene and Tropical Medicine, London, United Kingdom; Baylor College of Medicine, UNITED STATES

## Introduction

On May 22, 2001, a resolution passed during the 54th World Health Assembly (WHA 54.19) took an historic step toward reducing the morbidity and mortality associated with the world’s most common parasitic worm (helminth) infections [1]. Indeed, an estimated 1.45 billion individuals are infected with soil-transmitted helminths worldwide [2]. The soil-transmitted helminths primarily comprise hookworm (*Ancylostoma duodenale* and *Necator americanus*), roundworm (*Ascaris lumbricoides*), and whipworm (*Trichuris trichiura*). Taken together, soil-transmitted helminthiasis accounts for a global burden of over 3.3 million disability-adjusted life years [3] and is associated with anemia [4], malnutrition [5], and impaired physical and cognitive development [6–9]. As the primary recommendation to eliminate soil-transmitted helminthiasis as a public health problem, WHA 54.19 called for improved water and sanitation to reduce transmission and urged that 3 high-risk groups receive regular treatment with anthelmintic drugs: preschool-aged children (PSAC), school-aged children (SAC), and women of reproductive age (WRA) [1].

During the decade 2001–2010, however, soil-transmitted helminthiasis control focused almost exclusively on preventive chemotherapy targeting SAC through the education sector, with a target of achieving at least 75% drug coverage in this population group by 2010 (Table 1). While this target was not reached [10], global efforts to address soil-transmitted helminthiasis were renewed in 2011, when several high-level meetings took place and their reports published in subsequent years. The “Roadmap on Neglected Tropical Diseases” was published first, reiterating the 75% preventive chemotherapy coverage target for PSAC and SAC [11]. This roadmap inspired 22 partners from public and private sectors to endorse the London Declaration on Neglected Tropical Diseases [12] and called on all partners to sustain and expand programs to achieve the 2020 goals outlined in the roadmap. Subsequently, a specific strategic plan for soil-transmitted helminthiasis was published, also in 2012, in which, on top of the 75% target for coverage, the additional target of reducing moderate- and heavy-intensity infections (defined as the number of helminth eggs excreted by an individual exceeding a preset, species-specific threshold, used as a proxy for worm burden) to less than 1% among SAC was affirmed [13]. With 2020 on the horizon, we are well into the second decade post-WHA 54.19. Major challenges remain. Among others, these include (1) the need to maximize the impact of pharmaceutic donations of anthelmintic drugs, (2) the need to clarify targets to guide monitoring efforts moving forward, and (3) the need to take into account recent successes of the Global Program to Eliminate Lymphatic Filariasis (GPELF). The latter challenge results in the consequent scaling down of community-based control interventions, thus reducing the ancillary benefits of this strategy on soil-transmitted helminthiasis [14]. A complete transition from a lymphatic filariasis elimination program to a soil-transmitted helminthiasis control program will have important consequences so that efforts to ensure that all risk groups for soil-transmitted helminthiasis will be adequately covered need to be planned well in advance of the actual transition.

**Table 1:** Overview of the evolution of documents published by WHO pertaining to the control of soil-transmitted helminthiasis since 2001.

Year	Document	Goal	Risk group(s)	Controlling morbidity: specific targets	Parasitologic monitoring: specific targets
2001	WHA 54.19 [1]	“To sustain successful control activities in low-transmission areas in order to eliminate soil transmitted helminth infections as a public health problem, and to give high priority to implementing or intensifying control of soil transmitted helminth infections in areas of high transmission” (p. 1)	PSAC, SAC, WRA	• “Regular administration of chemotherapy to at least 75%, and up to 100%, of all school-age children at risk of morbidity by 2010” (p. 1)	Not mentioned
2002 (Second edition published in 2012)	Helminth Control in School-age Children [77]	“Reduce worm loads [in SAC] and keep them low” (p. 8)	SAC	• “Regular delivery of anthelminthic treatment to at least 75% of school-age children in endemic areas” (p. 8)	• “The proportion of children heavily infected has been reduced to less than 1% in 2–3 years” (p. 44) • “The proportion of children with morbidity resulting from STH [soil transmitted helminth] infection and/or schistosomiasis has been reduced to less than 1% in 5 years” (p. 44)
2012	WHO Strategic Plan 2011–2020 [13]	“Reduce morbidity from STH [soil-transmitted helminthiasis] in preschool-aged children (aged 1–4 years) and school-age children (aged 5–14 years) to a level below which it would not be considered a public health problem^1^” (p. 20)	PSAC, SAC	• “75–100% of children (SAC and PSAC) needing preventive chemotherapy worldwide have been treated [by 2020]” (pg. 29) • “100% of countries requiring preventive chemotherapy for STH [soil transmitted helminthiasis] have achieved 75% national coverage of SAC and PSAC [by 2020]” (p. 29)	• “Less than 1% of countries requiring preventive chemotherapy for STH [soil-transmitted helminthiasis] have infection of high or moderate intensity [by 2020]” (p. 29) • “100% of countries requiring preventive chemotherapy for STH [soil-transmitted helminthiasis] regularly assess intensity of infections in sentinel sites [by 2020]” (p. 29)
2012	WHO 2020 Roadmap on Neglected Tropical Diseases [11]	Soil-transmitted helminthiasis is included under diseases listed with “targets and milestones for control of neglected tropical diseases, 2015–2020” (p. 19)	PSAC, SAC	• “75% of preschool and school-aged children in need of treatment are regularly treated [by 2020]” (p. 5) • “75% coverage achieved in preschool and school-aged children in 100% of countries [by 2020]” (p. 19)	Not mentioned

**Abbreviations:** PSAC, preschool-aged children; SAC, school-aged children; WHA, World Health Assembly; WHO, World Health Organization; WRA, women of reproductive age.

^1^ Soil-transmitted helminthiasis is considered a public health problem when the prevalence of soil-transmitted helminth infection of moderate and heavy intensity among SAC is over 1%.

If the goal of eliminating soil-transmitted helminthiasis as a public health problem is to be achieved, it is important to proactively review and address gaps in disease control programs. The Soil-Transmitted Helminthiasis Advisory Committee (subsequently termed “the Committee,” established in 2012, as the successor of the Mebendazole Advisory Committee that was launched in 2006) is an independent group of experts that holds an annual meeting to assess challenges and review progress made in soil-transmitted helminthiasis control, including operational research, monitoring, and evaluation, and to deliberate on next steps. The Committee makes recommendations to address technical and scientific challenges and provides advice to members of the Soil-Transmitted Helminthiasis Coalition and the World Health Organization (WHO) Strategic and Technical Advisory Group (STAG). On October 18–19, 2016, the Committee convened for 2 days in Basel, Switzerland, to review and discuss advances in operational research, anthelmintic treatment options, and diagnostic tools and strategies. Furthermore, programmatic and strategic challenges in global control efforts were debated. Here, we present the recommendations arising from this meeting and highlight challenges and potential solutions on the road toward the 2020 goal of soil-transmitted helminthiasis control and elimination and beyond.

## Controlling soil-transmitted helminthiasis morbidity

### Progress and challenges

The Soil-Transmitted Helminthiasis Strategic Plan 2011–2020 [13] has outlined 4 primary milestones for global control of soil-transmitted helminthiasis: (1) 100% of countries requiring preventive chemotherapy for soil-transmitted helminthiasis have achieved 75% national coverage of PSAC and SAC, (2) these countries regularly assess intensity of soil-transmitted helminth infections in sentinel sites, (3) less than 1% of countries requiring preventive chemotherapy for soil-transmitted helminthiasis have infection of moderate or high or intensity by 2020, and (4) 75%–100% of PSAC and SAC needing preventive chemotherapy worldwide have been treated.

In 2016, the Weekly Epidemiological Record (WER) reported the global progress toward milestones 1 and 4, indicating that <30% of countries requiring preventive chemotherapy for soil-transmitted helminthiasis had achieved the 75% national coverage target for PSAC and SAC and that 48% of PSAC and 65% of SAC needing preventive chemotherapy worldwide had received treatment [15]. It was not possible to report on either milestone 2 or 3 because there were no publicly available data to review whether sentinel surveillance or parasitologic monitoring was being implemented in the endemic countries. Based on current progress toward milestones 1 and 4, it is anticipated that these may potentially be achieved by 2020 (at least for SAC), whereas milestones 2 and 3 are less likely to be reached by 2020. Using the London Declaration Scorecard (http://unitingtocombatntds.org/reports/5th-report/), the Committee noted that milestones for country reporting on coverage and parasitologic monitoring were lagging.

In order to bring progress toward milestones 2 and 3 on track, the Committee suggests that barriers to program implementation be acknowledged and that technical support be provided to countries struggling to reach the 75% national coverage targets. As the year 2020 nears, there is a pressing need for the global community to consider a serious recommitment to milestones 2 and 3 and to work together to improve parasitologic assessment in affected countries. The London Declaration Scorecard remains a useful tool in monitoring progress toward these milestones, but the Committee recommends that the Scorecard be updated to include water, sanitation, and hygiene (WASH) indicators to be aligned with the United Nations Sustainable Development Goals (SDGs; http://www.un.org/sustainabledevelopment/sustainable-development-goals/) and that treatment be expanded to include other at-risk groups, most importantly WRA. Beyond being explicitly called for in WHA 54.19, these additional measures will likely be needed to accelerate elimination of soil-transmitted helminthiasis as a public health problem in children [16–20]. In this context, a robust, integrated, and regularly updated global surveillance platform is needed. Ideally, this platform could also be used for schistosomiasis and other neglected tropical diseases [21,22].

The Committee also recognizes the unique contribution of GPELF to concurrently control soil-transmitted helminthiasis–related morbidity. Launched in 2002 by WHO, GPELF has successfully treated an estimated 36 million PSAC and 139 million SAC in 2015 with combination preventive chemotherapy that included albendazole [23], one of the two donated anthelmintic drugs widely used against soil-transmitted helminthiasis [24]. The GPELF community-based delivery platform reaches at-risk groups outside of the school setting and through the coadministration of 2 drugs with a different mechanism of action (e.g., albendazole and ivermectin) that, as shown for animal helminthiasis, are likely to reduce the risk of resistance [25,26]. It follows that GPELF has enhanced the coverage and effectiveness of soil-transmitted helminthiasis control activities in many countries. However, there is an immediate risk of losing this delivery infrastructure as the GPELF achieves its goal and as national governments and donors scale down or discontinue their support for the program. Hence, without a strategic transition plan in place, communities that used to benefit from lymphatic filariasis control activities run the risk of undermining the gains already made for soil-transmitted helminthiasis control once GPELF is discontinued. The Committee therefore proposes that (1) a parasitologic assessment be first conducted in areas where termination of lymphatic filariasis control activities is being contemplated and that (2) WHO convenes a technical working group to develop a decision algorithm for countries on when and how to implement a lymphatic filariasis–soil-transmitted helminthiasis transition. Such an algorithm will be especially important in areas where there are no clear alternatives for continuing preventive chemotherapy for soil-transmitted helminthiasis among SAC and other at-risk groups [27,28].

Clearly, eliminating soil-transmitted helminthiasis as a public health problem has to go beyond preventive chemotherapy for SAC alone, as other groups at risk also serve as a reservoir of infection, e.g., hookworm infections frequently predominate in adult populations [29]. Coverage of preventive chemotherapy for PSAC continues to lag behind the coverage for SAC; to date, there is no regular preventive chemotherapy program against soil-transmitted helminthiasis for WRA (although, some countries have developed such programs specifically for pregnant women). To address these gaps, we recommend that specific guidelines for the treatment of PSAC and WRA be developed and validated under the lead of WHO, including a regular reporting mechanism for the treatment coverage in these groups. Taken together, there is a need for a robust, integrated, and regularly updated global neglected tropical disease surveillance platform to include interactive preventive chemotherapy data (http://apps.who.int/gho/cabinet/pc.jsp) and georeferenced survey and intervention data (https://www.gntd.org) [22,30].

### Treatment options and new developments

In comparison to other classes of anti-infective drugs such as antibiotics, the number of anthelmintics that are used in human medicine is limited, and there have been far fewer innovations or new compounds developed to broaden the pharmacologic armamentarium [24,31–34]. In this context, several important challenges for soil-transmitted helminthiasis control need to be highlighted, i.e., (1) the development of a new and rapidly disintegrating, chewable formulation of mebendazole for PSAC; (2) the introduction of combination treatment approaches for soil-transmitted helminthiasis; (3) the limited understanding of resistance development to anthelmintic drugs in human soil-transmitted helminthiasis; and (4) careful considerations pertaining to the continuing role of pharmaceutic drug donations in the post-2020 agenda.

Albendazole and mebendazole, both benzimidazole drugs, are widely used in preventive chemotherapy programs targeting soil-transmitted helminthiasis worldwide. Their anthelmintic properties differ slightly, with albendazole being more active against hookworm [31,35]. Of note, the efficacy of both compounds against *T*. *trichiura* is unsatisfactory, and low cure rates of single-dose administration have also been reported for hookworm infection, in particular if mebendazole is used. Other factors, such as suboptimal dissolution of the tablets, may further decrease their therapeutic effects [36]. In addition, PSAC, especially those less than 3 years of age, have difficulty chewing and swallowing the relatively large tablets [37], and several deaths have been caused by aspiration and choking [38]. Hence, the Committee welcomes the recent approval of a new, rapidly disintegrating chewable formulation of mebendazole. The drug’s efficacy and tolerability have been shown in a study conducted in Ethiopia and Rwanda [37], and the drug received approval by the United States Food and Drug Administration (FDA) on October 19, 2016. We now advocate that access to this new formulation be provided in endemic areas, particularly for preventive chemotherapy targeting PSAC.

It has been suggested that widespread use of monotherapy might facilitate the development of anthelmintic drug resistance [39–44]. Hence, as for other chronic infections (e.g., tuberculosis, human immune deficiency virus [HIV], and malaria), combination therapy against soil-transmitted helminthiasis might decrease this risk and could enhance efficacy [25]. Moreover, the need for combination therapy is further supported by coendemicity of multiple helminth infections. Indeed, in many endemic settings, infections due to *A*. *lumbricoides*, hookworm, and *T*. *trichiura* co-occur. Recent studies reported an improved drug efficacy if a combination of albendazole plus either ivermectin or oxantel pamoate was administered [45–47]. An overview of currently used anthelmintics and promising drug combinations is presented in Table 2. Logistically, it would be desirable to develop coformulations of drug combinations that could be distributed as a single tablet (such combinations are readily available for other conditions, e.g., arterial hypertension, HIV/AIDS, and tuberculosis), but pharmacologic challenges need to be resolved before this approach becomes feasible in daily practice. While combination therapy may temporarily lower the resistance pressure, there is also a clear need for new anthelmintic drugs to ensure access to efficacious treatment options in the future. Additionally, ongoing research on developing anthelmintic vaccines can also provide important additional control tools, which may be integrated into future control strategies. The Committee urges that further studies be conducted to identify the most promising drug combinations for preventive chemotherapy against soil-transmitted helminthiasis. It applauds new funding granted by the Bill & Melinda Gates Foundation that addresses some of these issues, which has already resulted in the addition of the albendazole plus ivermectin combination to the WHO Model List of Essential Medicines [48]. Additionally, we welcome ongoing research projects that will strengthen the monitoring and surveillance of drug efficacy and anthelmintic resistance in soil-transmitted helminthiasis control programs (e.g., the Bill & Melinda Gates Foundation-funded STARWORMS [Stop Anthelminthic Resistant WORMS] project; http://www.starworms.org).

**Table 2:** Efficacy of anthelmintic drugs used for the treatment of soil-transmitted helminthiasis and recent evidence from clinical trials pertaining to 3 drug combinations.

Single drug	Spectrum of activity against soil-transmitted helminthiasis			Reference
	*Ascaris lumbricoides*	Hookworm	*Trichuris trichiura*	
Albendazole	+++	++	+	[24]
Levamisole	+++	+	+	[34]
Mebendazole	+++	+	+	[78]
Pyrantel pamoate	+++	+	+	[34]
**Drug combinations**	**Available evidence from clinical trials**			**Reference(s)**
Albendazole + ivermectin	• Improved activity against *T*.* trichiura* • Potentially less reinfection than after monotherapy with albendazole alone • Ivermectin is active against *Strongyloides stercoralis*			[46,47,79]
Albendazole + oxantel pamoate	• Highest activity of tested drug combinations against *T*.* trichiura* • Less reinfection than after monotherapy with albendazole alone			[45, 47]
Tribendimidine + oxantel pamoate or ivermectin	• Noninferior efficacy profile to albendazole + oxantel pamoate • Ivermectin is active against *S*.* stercoralis*			[80]

Abbreviations: +++, excellent efficacy (cure rates >90%); ++, moderate efficacy (50%–90%); +, low efficacy (<50%).

Experience and lessons from preventive chemotherapy programs targeting millions of mainly SAC were only possible through drug donations by the manufacturing pharmaceutic industry. However, it is important to note that a long-lasting, durable strategy for soil-transmitted helminthiasis control or even elimination cannot solely rely on such drug donation programs. As generic deworming drugs will become increasingly important in the future, particularly in the post-2020 agenda, the Committee urges WHO to encourage prequalification of the manufacturers of these drugs.

## Parasitologic monitoring

### Progress and challenges

Survey methods currently endorsed by WHO to assess the prevalence of any soil-transmitted helminth infection are not designed to determine whether or not the goal of eliminating soil-transmitted helminthiasis as a public health problem in children has been achieved. Hence, there is a need to develop a new survey design that (1) is sufficiently powered to assess if the prevalence of moderate- or heavy-intensity infections falls below 1% and (2) is feasible and affordable, considering the limited resources and capacity of national soil-transmitted helminthiasis control programs. Any new methodology being proposed should enable the measurement of prevalence of soil-transmitted helminth infection in SAC, PSAC, WRA, and other risk groups, providing a more complete picture of the burden of soil-transmitted helminthiasis in the entire community [49]. The Committee urges WHO to spearhead discussions with stakeholders to refine this survey methodology and, after successful field validation, to support and endorse its use so that it can be adopted by countries before 2020. At the same time, areas where the prevalence of soil-transmitted helminth infection continues to be high despite several years of preventive chemotherapy would warrant further investigation as these serve as potential indicators of previously unrecognized programmatic challenges.

### Diagnostic methods and new developments

Accurate diagnostic techniques for soil-transmitted helminth infection are of paramount importance in settings where the overall prevalence is low and, even more importantly, where the majority of infections are of light intensity. Indeed, different diagnostic techniques are required at different stages of helminthiasis control programs, e.g., to prove elimination as a public health problem or to document an interruption of transmission. The detection limit of most diagnostic techniques decreases considerably in such areas, and techniques with a higher sensitivity are required for an accurate assessment of remaining foci of endemicity [50]. It has recently been argued that the development of new and more sensitive diagnostic techniques has been slowed down by the strong focus on drug coverage rather than parasitologic monitoring in most soil-transmitted helminthiasis control programs [51]. The development of several new techniques, many of which are not based on stool microscopy, are encouraging. Their different features and characteristics are summarized in Table 3.

**Table 3:** Brief characterization of the Kato–Katz technique and selected other diagnostic developments for detection of soil-transmitted helminths, which might potentially be used in soil-transmitted helminthiasis control programs and epidemiologic surveys.

Diagnostic technique	Principle	Characteristics	Reference(s)
Kato-Katz thick-smear	• Smear-based stool microscopy • Detection is based on the morphology of eggs	• WHO-recommended standard technique for epidemiologic surveys • Examination of 41.7 mg of stool Simultaneous detection of soil-transmitted helminth and *Schistosoma* eggs • Relatively simple to perform • Sensitivity dependent on infection intensity (unreliable in populations with a low prevalence and light infection intensity) and number of thick smears prepared • Hookworm eggs are not reliably detected after 30–60 min	[81–83]
Mini-FLOTAC	• Flotation-based stool microscopy • Detection is based on the morphology of eggs, larvae, and cysts	• Further development of the original FLOTAC technique, without need for centrifugation (hence, no electricity required) • One of the WHO-recommended methods in transmission assessment surveys • Examination of 100 mg of stool • Simultaneous detection of helminth eggs (soil-transmitted helminths and *Schistosoma* spp.), larvae, and intestinal protozoa (*Giardia intestinalis*, *Entamoeba* spp.) depends on the choice of flotation solution	[84–86]
FECPAK^G2^	• Flotation-based stool microscopy • Detection is based on the morphology of the eggs	• Initially developed for the diagnosis of animal soil-transmitted helminths but currently being optimized and validated for human soil-transmitted helminths • Currently only available for the diagnosis of veterinary soil-transmitted helminths • Examination of approximately 3 g of stool • Allows accumulation of eggs in 1 microscopic view, digital images are taken by an autonomously operating digital picture microscope, images are sent via e-mail for analysis elsewhere • Waives the need for laboratory infrastructure in epidemiologic studies • Holds promise for quality assurance activities	[87–89]
PCR	• Nucleic acid-based molecular technique • Detection of specific nucleic acid sequences of target pathogens	• Allows differentiation of zoonotic and human soil-transmitted helminth species • Technically difficult, requires well-equipped laboratories with constant power supply and experienced laboratory technicians • No well-established quality assurance system for PCR diagnostics for soil-transmitted helminth infection is currently in place • Concurrent detection of several helminth and intestinal protozoa species possible	[90]
RPA	• Nucleic acid-based molecular technique • Detection of specific nucleic acid sequences of target pathogens	• Highly sensitive and specific detection of pathogen-specific nucleic acids • No need for a thermal cycler; hence, no need for electricity • Available for intestinal protozoa, schistosomiasis and fascioliasis, but not (yet) for soil-transmitted helminthiasis	[91,92]
LAMP	• Nucleic acid-based molecular technique • Detection of specific nucleic acid sequences of target pathogens	• Characteristics similar to RPA • Several published studies reporting high sensitivity and specificity for detection of soil-transmitted helminth species	[93,94]

**Abbreviations:** LAMP, loop-mediated isothermal amplification procedure; PCR, polymerase chain reaction; RPA, recombinase polymerase amplification.

As soil-transmitted helminthiasis control efforts evolve, diagnostic techniques must also be further developed to ensure that their application is feasible and that the reported results are accurate. In areas where preventive chemotherapy has been employed for many years, conventional techniques based on stool microscopy alone might fail to demonstrate the persistence of light-intensity infections [50,52]. In such instances, more sensitive molecular methods such as stool-based polymerase chain reaction (PCR) assays may depict the “true” situation more accurately. In settings of a very low prevalence of soil-transmitted helminth infection, pooling of stool specimens and subsequent PCR examination might be a promising method to detect and monitor areas of ongoing transmission, although costs and required logistic infrastructure require elaboration [53,54].

Additionally, a transition away from stool specimen analysis to, for example, blood- or urine-based tests for antigen or antibody detection might further enhance the accurate diagnosis of soil-transmitted helminthiasis [55,56]. For the aforementioned methods, in particular the molecular techniques, adequate specimen preservation, simplified nucleic acid extraction, and quality assurance systems are crucial [57]. This is important as many laboratories use a wide variety of in-house PCR techniques for detection of helminths, but target genes and techniques used differ considerably. Following 2 expert meetings held in Ghent, Belgium and Annecy, France in mid-2016, recommendations were made to develop target product profiles for different use-cases and to prepare field sites for large-scale validation studies of helminth PCR techniques. The Committee encourages rigorous, multicenter evaluations and strategic developments for large-scale application and setups for external quality assurance systems of such PCR techniques in the field.

#### Evolution of soil-transmitted helminthiasis control: Clarifying the goals

When reviewing the different documents pertaining to the global strategy against soil-transmitted helminthiasis (Table 1), the roadmap in particular fails to mention previous targets with respect to coverage and morbidity reduction. The Committee therefore urges WHO to ensure that all relevant future documentation reaffirm both morbidity control and parasitologic monitoring targets in order to allay any confusion or programmatic concerns. While the 2020 target of 75% drug coverage may be in reach for SAC—and perhaps PSAC [15]—in certain countries, it is likely that elimination of soil-transmitted helminthiasis as a public health problem will remain a challenge. In our view, rigorous parasitologic monitoring is required. The current strategy and the strong emphasis on drug coverage targets offer only indirect endpoints for national soil-transmitted helminthiasis control programs. A comparative assessment of the different strategies for soil-transmitted helminthiasis control and its arising implications for national control programs is presented in Table 4. The Committee supports a recent call [58] and stresses the importance for clarifying the goals of soil-transmitted helminthiasis control strategies.

**Table 4:** Key characteristics of 3 different strategies pertaining to future soil-transmitted helminthiasis control efforts.

Goal	Priority Indicator	Implications
**Original (and current) strategy:** Deworm high-risk groups	At least 75% of children in need of treatment are regularly treated	• **Endpoint:** measurable endpoint but no indicator of morbidity, no stopping strategy • **Parasitologic monitoring:** limited monitoring required • **Platform:** school or child health day platforms may be adequate **• Water, sanitation, and hygiene:** integration advocated • **Cost:** least expensive • **Research:** little operational research required
**Revised strategy:** Elimination of soil-transmitted helminthiasis as a public health problem	Less than 1% moderate or heavy infection intensity prevalence in all risk groups	• **Endpoint:** measurable endpoint, indirect indicator of morbidity • **Parasitologic monitoring:** intense monitoring required • **Platform:** integrated or community-based platform may be required • **Water, sanitation, and hygiene:** intense integration required • **Cost:** more expensive • **Research:** operational research required
**Ambitious strategy:** Interruption of soil-transmitted helminth transmission	Less than 1% overall soil-transmitted helminth infection prevalence in all risk groups	• **Endpoint:** measurable endpoint, indirect indicator of morbidity • **Parasitologic monitoring:** intense monitoring and evaluation required • **Platform:** integrated or community-based platform required • **Water, sanitation, and hygiene:** more intense integration required • **Cost:** most expensive • **Research:** operational research required

Several specific aspects underscore the importance of the choice of one common strategy for soil-transmitted helminthiasis control. For example, it is generally acknowledged that soil-transmitted helminth infection negatively impacts health, especially when infection intensity is high; that safe and effective anthelmintic drugs are available to reduce morbidity; and that preventive chemotherapy is an effective way to reach those at risk. However, a recent Cochrane review [59] and a systematic review with network meta-analysis [60], while criticized for their methodologic limitations and other concerns [61,62], challenged some of the portrayed beneficial effects. The Committee advocates for rigorous parasitologic monitoring after several rounds of preventive chemotherapy to assess the reduced burden of moderate- and high-intensity infections associated with morbidity, enabling a more accurate quantification of the likely health benefits of deworming. Indeed, a return from a treatment coverage target to the original goal of eliminating soil-transmitted helminthiasis as a public health problem, and the prioritization of parasitologic monitoring would offer a measurable and direct endpoint for national programs (i.e., less than 1% moderate or heavy infection intensity prevalence in all risk groups). At present, it remains unclear whether this goal can be reached through preventive chemotherapy alone. It is also important to note that sustaining the gains against soil-transmitted helminthiasis made possible by community-based GPELF programs will not be feasible without taking the goal of eliminating soil-transmitted helminthiasis as a public health problem seriously, as monitoring is needed to guide the planning of a transition from lymphatic filariasis elimination to soil-transmitted helminthiasis control. At present, drug coverage in the 3 identified high-risk groups alone is too often the main focus. It may be that other at-risk groups (e.g., adolescents and adults) constitute an important reservoir of transmission, which may need specific control efforts. Accounting for these populations will more accurately reflect the success of a control program [63].

The interruption of soil-transmitted helminthiasis transmission is a topic of growing interest [64,65]. History shows that sustained control efforts, coupled with economic development, can lead to transmission interruption in different parts of the world [66–68]. However, the attributable preventive fraction of different control strategies for successful transmission interruption is difficult to assess, and many experts emphasized that urbanization, economic development, and improved hygiene are more important factors than repeated anthelmintic treatment because rapid reinfection occurs frequently in highly endemic areas. As it is unlikely that such rapid and sustained economic developments will occur anytime soon in many of the most affected low-income countries, this underscores the importance of additional tools for control efforts. Yet, mathematical modeling also suggests that interruption of transmission is feasible in soil-transmitted helminthiasis–endemic settings that are characterized by low-infection intensities [65]. The burden of soil-transmitted helminthiasis varies across settings. A multifaceted, intersectoral approach along with appropriate delivery platforms is needed to achieve the ambitious goal of interrupting soil-transmitted helminthiasis transmission at a local level. WHA 54.19 originally called for improved access to WASH through intersectoral collaboration. Growing evidence supports that call [69] and further pleas for WASH integration into neglected tropical disease control programs have been re-emphasized [70]. While WHO has begun to integrate WASH into its global neglected tropical disease control strategy [71], guidelines are needed for the implementation of specific WASH interventions into soil-transmitted helminthiasis control strategies, similar to that of the “SAFE” strategy targeting trachoma [72–74].

Vast resources have been contributed to sustain and expand programs for the 10 neglected tropical diseases highlighted in the London Declaration to be eradicated, eliminated, or controlled by 2020. While many programs registered success due to this collaborative effort [75,76], guidelines for the evaluation of soil-transmitted helminthiasis control programs need to be strengthened. The Committee advocates that parasitologic surveys need to be performed after several years of preventive chemotherapy to document whether or not the goals set by WHO (i.e., those pertaining to infection intensity) have been achieved. Suitable approaches (e.g., adequately powered surveys) need to be included in a revised WHO strategic plan. The key recommendations put forth by the Committee are summarized in Box 1.

**Box 1. Key items, related challenges, and recommendations to all stakeholders put forth by the Soil-Transmitted Helminthiasis Advisory Committee (“the Committee”) at the 2016 annual meeting with regard to global control efforts for soil-transmitted helminthiasis**

**Key item 1: Elimination of soil-transmitted helminthiasis as a public health problem**

Challenge: While elimination as a public health problem is clearly defined (<1% prevalence of moderate- or heavy-intensity infections for any soil-transmitted helminth species in a distinct geographic area), there is disagreement on the tools needed to achieve and to assess elimination.

Recommendation: The Committee supports this definition of soil-transmitted helminthiasis elimination and urges all stakeholders to develop a common strategy on how to achieve this goal.

**Key item 2: Parasitologic monitoring of control efforts**

Challenge: Accurate parasitologic studies are not carried out on a regular basis in many endemic areas. There is no agreement on a sampling design to determine if set targets have been reached.

Recommendation: Parasitologic monitoring in endemic countries is essential for assessing progress toward the elimination goal. WHO should develop and support a sampling design that is powered enough to determine if the goal of <1% prevalence of moderate- or heavy-intensity infection has been reached and affordable and relatively easy to implement given the limited resources available to, and capacity of, national control programs. The reporting of age- and sex-disaggregated data should be emphasized.

**Key item 3: Anthelmintic treatment coverage of at-risk groups**

Challenge: Anthelmintic treatment rates for preschool-aged children (PSAC) lag behind coverage rates reported for school-aged children (SAC), while most women of reproductive age (WRA) remain untreated amid scale-up efforts in high-burden countries.

Recommendation: Conduct operational research to identify challenges for coverage of PSAC and WRA and use findings for developing new guidelines for these risk groups.

**Key item 4: Reporting on treatment coverage and data sharing on subnational level**

Challenge: WRA are among the at-risk groups for whom deworming is recommended, but treatment coverage data are not reported. For all groups receiving treatment, it would be more informative to have regional- and/or district-specific coverage rates in addition to national estimates.

Recommendation: WHO should improve reporting of treatment coverage rates by inclusion of WRA in the regular updates on soil-transmitted helminthiasis published in the Weekly Epidemiological Record (WER). Additionally, WHO can provide a platform where subnational-level data on drug coverage in soil-transmitted helminthiasis–endemic districts should be shared whenever available. It is further suggested that WHO report the proportion of soil-transmitted helminthiasis–endemic districts (globally and by country) that have reached at least 75% coverage.

**Key item 5: Water, sanitation, and hygiene (WASH)**

Challenge: WASH is essential for soil-transmitted helminthiasis elimination as a public health problem and more investment is needed to include this important component into control efforts.

Recommendation: Long-term investments for soil-transmitted helminthiasis–specific WASH are needed, and WASH indicators should be included in the London Declaration Scorecard that align with the 2030 Sustainable Development Goal (SDG) 6, that is to “ensure availability and sustainable management of water and sanitation for all”.

**Key item 6: Transition from lymphatic filariasis elimination to soil-transmitted helminthiasis control**

Challenge: Many ancillary benefits of the Global Program for Eliminating Lymphatic Filariasis (GPELF) with regard to soil-transmitted helminthiasis might be lost if GPELF is scaled down.

Recommendation: Design an effective transition strategy and conduct operational research to identify and promote the policy frameworks, capacity building, planning, and intersectoral collaboration needed to sustain the contributions of the lymphatic filariasis program to progress made in soil-transmitted helminthiasis control.

**Key item 7: Clinical morbidity due to soil-transmitted helminthiasis**

Challenge: Soil-transmitted helminths cause primarily chronic, subtle morbidity, which is difficult to assess. For an accurate estimation of the attributable disease burden, clinical studies are needed.

Recommendation: Gather scientific evidence pertaining to clinical morbidity due to soil-transmitted helminthiasis and address how soil-transmitted helminthiasis control activities may lead to a measurable decrease of morbidity in endemic areas.

**Key item 8: Laboratory diagnosis of soil-transmitted helminthiasis**

Challenge: The global implementation of standard diagnostic tools based on microscopy (i.e., Kato-Katz technique) allowed comparison between countries, but these are not sensitive enough to be used in settings where elimination of soil-transmitted helminthiasis seems feasible and where infection intensities are low.

Recommendation: Continue the development and validation of recent advances in PCR-based diagnostics for soil-transmitted helminthiasis and conduct rigorous, multisite evaluations, and strategic developments for larger-scale application in the field.

**Key item 9: Combination therapy for soil-transmitted helminthiasis**

Challenge: Preventive chemotherapy programs have increased considerably worldwide. Yet no single drug is equally effective in achieving satisfactory cure rates for the major soil-transmitted helminths, and drug resistance is expected to arise and further decrease the efficacy of available treatment options.

Recommendation: The Committee appreciates both the efforts and challenges to developing coformulations of anthelmintic drugs and supports research initiatives that assess the safety and efficacy of various combination therapies in order to identify which combination is best suited for scaling up to achieve a maximum impact.

**Key item 10: Ownership of soil-transmitted helminthiasis control programs**

Challenge: Drug donation is the cornerstone of current soil-transmitted helminthiasis control efforts in many areas, but this is not a sustainable solution without a long-term perspective.

Recommendation: Country ownership of soil-transmitted helminthiasis prevention and control programs is paramount, and future efforts should not rely on drug donation alone. Generic deworming drugs will become increasingly important post-2020, and WHO should encourage the prequalification of the manufacturers of these drugs.

**Key item 11: Foci of continued soil-transmitted helminthiasis transmission**

Challenge: Guidance is required on how to deal with foci of ongoing transmission in areas where a regular preventive chemotherapy program is no longer being implemented.

Recommendation: WHO should develop or endorse decision-making guidelines for program managers to identify and respond to foci of “unexpectedly high” soil-transmitted helminthiasis transmission post-preventive chemotherapy program.
